# The gut microbiome in lung cancer: from pathogenesis to precision therapy

**DOI:** 10.3389/fmicb.2025.1606684

**Published:** 2025-08-20

**Authors:** Miao Shi, Long-Fei Wang, Wen-Tao Hu, Zhi-Gang Liang

**Affiliations:** Department of Thoracic Surgery, First Affiliated Hospital of Ningbo University, Ningbo, China

**Keywords:** gut microbiome, gut-lung axis, lung cancer, immunotherapy, microbial biomarkers

## Abstract

The gut microbiome has emerged as a key modulator of immune responses and treatment efficacy in oncology. Growing evidence links gut dysbiosis to resistance against immune checkpoint inhibitors (ICIs) in advanced cancers, prompting exploration of the gut-lung axis—a bidirectional network connecting intestinal microbiota with pulmonary health. Given lung cancer’s status as the leading cause of cancer mortality worldwide, understanding this axis holds significant therapeutic potential. This review synthesizes current knowledge on gut microbiota’s role in lung cancer development, diagnosis, and treatment. We highlight microbial signatures predictive of disease and therapy response, discuss microbiota-targeted interventions (e.g., probiotics, Fecal Microbiota Transplantation), and elucidate mechanistic insights into microbial-immune crosstalk. Finally, we outline future directions for leveraging the gut microbiome in personalized lung cancer management.

## 1 Introduction

Lung cancer maintains its position as the leading cause of cancer-related mortality worldwide, accounting for approximately 2.2 million new cases and 1.8 million deaths annually (Sung et al., 2021). Histologically, lung cancer is broadly classified into small cell lung carcinoma and non-small cell lung carcinoma (NSCLC), with the latter accounting for over 85% of all cases (Sung et al., 2021). Globally, the most common histological subtypes of NSCLC are adenocarcinoma (40%) and squamous cell carcinoma (25%) (Su et al., 2025). Smoking is a well-established risk factor for lung cancer, while other risk factors include lifestyle and environmental exposures such as biomass fuel exposure, occupational hazards, and air pollution (Leiter et al., 2023). Additionally, genetic predisposition and gender are significant risk factors that cannot be overlooked (Jemal et al., 2018). Despite therapeutic advancements including radiotherapy, chemotherapy, immunotherapy, and surgical interventions, clinical outcomes remain suboptimal, with persistently low survival rates and substantial treatment-related toxicities underscoring the urgent need for innovative approaches (Lahiri et al., 2023). In this context, the conceptual framework of the “gut-lung axis” has gained considerable traction, proposing a sophisticated bidirectional communication network between intestinal microbiota and pulmonary physiology mediated through integrated immune, neural, and metabolic pathways. The gut microbiota exerts systemic immunomodulatory effects through microbial metabolite production and immune cell priming, while pulmonary inflammatory responses can reciprocally influence gut microbial ecology via circulating cytokines and neuroendocrine signals. This paradigm-shifting understanding of gut-lung crosstalk has catalyzed new research directions in lung cancer therapeutics, suggesting that targeted manipulation of gut microbial communities may offer a promising strategy to enhance treatment efficacy and reduce adverse effects.

The gut microbiome has emerged as a pivotal regulator in tumor immunology, with mounting evidence establishing its critical role in modulating host immune responses to cancer therapy. Particularly compelling is the association between gut dysbiosis and primary resistance to immune checkpoint blockade (ICB) across multiple advanced malignancies (Simpson et al., 2023; Shi et al., 2023; Soularue et al., 2018). This relationship has been substantiated by extensive epidemiological investigations demonstrating that broad-spectrum antibiotic use, which disrupts gut microbial homeostasis, significantly impairs the clinical efficacy of anti-PD-1/PD-L1 antibodies in stage III/IV melanoma, as well as lung, renal, and bladder carcinomas (Salgia et al., 2020; Routy et al., 2023). Mechanistically, depletion of microbial diversity correlates with an immunologically “cold” tumor microenvironment characterized by insufficient cytotoxic T-cell infiltration. Emerging evidence suggests that strategic modulation of gut microbiota composition may potentiate antitumor immunity by enhancing T-cell activation and tumor cell phagocytosis, thereby opening novel therapeutic avenues for cancer management.

This review provides a comprehensive synthesis of contemporary research elucidating the multifaceted roles of gut microbiota in lung cancer pathogenesis, early detection, and therapeutic intervention. We will critically examine: (1) mechanistic insights into gut microbiome-mediated modulation of pulmonary tumor immunity; (2) microbial signatures associated with disease progression and treatment response; (3) current and emerging microbiota-targeted therapeutic strategies; and (4) future directions for translating these findings into clinical practice. By integrating cutting-edge research from microbiology, immunology, and oncology, this work aims to advance our understanding of the gut-lung axis and its therapeutic potential in lung cancer management.

## 2 The gut-lung axis: microbial regulation of pulmonary immunity and disease pathogenesis

Accumulating evidence has established the gut microbiota as a critical modulator of pulmonary health through the gut-lung axis—a sophisticated bidirectional communication network mediated by microbial metabolites, immune regulation, and neuroendocrine signaling (Huh and Veiga-Fernandes, 2020; Budden et al., 2017). In physiological conditions, commensal gut microbes maintain pulmonary immune homeostasis through multiple mechanisms: (1) production of immunomodulatory metabolites [e.g., short-chain fatty acids (SCFAs)] that enter systemic circulation and regulate lung immunity; (2) priming of dendritic cells and T cell populations that subsequently migrate to pulmonary tissues; and (3) maintenance of mucosal barrier integrity through tight junction protein modulation (Chakradhar, 2017; Wypych et al., 2019; Jeyanathan et al., 2022; Lin et al., 2025).

Disruption of gut microbial homeostasis precipitates a cascade of pathological events that compromise pulmonary defenses. Clinical studies have identified distinct gut microbiota signatures associated with specific respiratory diseases. The study found that children at high risk of asthma already exhibit dysbiosis of gut fungi, bacteria, and archaea before the onset of the disease (Barcik et al., 2020). In progress of asthma development, early-life gut dysbiosis characterized by elevated *Bacteroides* spp. (particularly *B. fragilis*) and anaerobic species, coupled with reduced microbial α-diversity and depletion of immunoprotective taxa including *Faecalibacterium prausnitzii* (a major butyrate producer), *Lachnospira* spp. (SCFA-producing genera), *Rothia mucilaginosa* (nitrate reducer), and *Veillonella parvula* (immunomodulatory species), significantly increases asthma risk (Lee-Sarwar et al., 2022; Depner et al., 2020). In a murine asthma model, the gut microbiota metabolite p-cresol sulfate was found to selectively suppress chemokine CL20 production in pulmonary epithelial cells by decoupling EGFR and TLR4 signaling, thereby reducing dendritic cell activation and exerting a protective effect against airway inflammation (Wypych et al., 2021).

In COPD patients, pro-inflammatory factors from the lungs migrate to the gastrointestinal tract via systemic circulation, promoting immune cell infiltration, epithelial barrier disruption, oxidative stress, hypoxia, and alterations in gut microbiota and metabolites. These intestinal impairments inhibit nutrient absorption, reduce antioxidant capacity, and weaken protective responses against pathogens and other environmental stimuli, thereby exacerbating COPD (Wang et al., 2023). The COPD-associated gut microbiome demonstrates marked expansion of pro-inflammatory *Muribaculaceae* (mucin-degrading specialists), *Desulfovibrionaceae* (sulfate-reducing bacteria), and specific *Lachnospiraceae* strains, which correlate with enhanced systemic inflammation and disease severity (Budden et al., 2024).

There is bidirectional immune crosstalk between pulmonary *Mycobacterium tuberculosis* and the gut microbiota. For instance, intestinal *Helicobacter* spp. infection can influence pulmonary *M. tuberculosis* infection and disease progression, while *M. tuberculosis* infection in the lungs may also alter the gut microbiota (Naidoo et al., 2019). Pulmonary Tuberculosis patients exhibit significant gut microbiota remodeling with a 45% increase in Actinobacteria (particularly *Bifidobacterium* spp.), 32% elevation in Proteobacteria (including *pathogenic Enterobacteriaceae*), and 28% reduction in Bacteroidetes—alterations associated with impaired IFN-γ production and compromised macrophage function (Luo et al., 2017). Concurrently, it was found that metabolites produced by pulmonary anaerobic bacteria (e.g., SCFAs) may modulate pulmonary immune responses and promote tuberculosis progression. Additionally, the loss of T-cell antigen epitopes in gut commensal non-tuberculous mycobacteria was shown to increase the risk of patient relapse (Naidoo et al., 2019). Beyond the aforementioned diseases, the relationship between lung cancer and gut microbiota has emerged as a research hotspot in recent years.

## 3 Alterations in gut microbiota composition and metabolic profile in lung cancer patients

Current research demonstrates that both intestinal and extraintestinal tumor development can induce significant pathological changes in the ileal mucosa, including mucosal atrophy and villous microvascular constriction mediated by sympathetic nervous system regulation of cholinergic signaling pathways. The onset of tumorigenesis triggers rapid secretion of REG3γ from ileal epithelial cells, causing transient increases in intestinal barrier permeability that ultimately result in substantial and long-lasting microbial imbalance, predominantly characterized by overgrowth of Gram-positive Clostridium species (Yonekura et al., 2022). Investigations into gut microbial diversity in lung cancer patients have yielded somewhat variable findings, yet the majority of studies indicate that both Shannon and Chao diversity indices in these patients remain largely comparable to those observed in healthy controls, suggesting minimal differences in overall microbial richness and diversity between the two groups (Zheng et al., 2020; Lim et al., 2021; Zhao et al., 2021; Qian et al., 2022).

Notwithstanding the preserved overall microbial diversity, lung cancer patients exhibit profound alterations in specific microbial taxa across multiple taxonomic levels. Examination at the family taxonomic rank reveals marked increases in *Ruminococcus*, *Enterobacteriaceae*, and *Lachnospiraceae* within the fecal microbiota of lung cancer patients, contrasted by significant depletion of beneficial genera including *Faecalibacterium* spp.*, Streptococcus* spp.*, Bifidobacterium* spp.*, and Veillonella* spp. (Zheng et al., 2020). These compositional changes extend to the genus level, where lung cancer patients demonstrate selective enrichment of unclassified genera within *Enterobacteriaceae* and *Lachnospiraceae* families as well as *Ruminococcus* spp. species, while experiencing notable decreases in the relative abundance of health-associated genera such as *Faecalibacterium* spp.*, Streptococcus* spp.*, Bifidobacterium* spp.*, and Veillonella* spp. (Zheng et al., 2020). Some studies comparing sequencing data from fecal samples of 41 lung cancer patients and 40 healthy volunteers found that at the genus level, the abundances of *Actinomyces* spp., *Veillonella* spp., *Megasphaera* spp., *Enterococcus* spp., and *Clostridium* spp. were higher in lung cancer patients than in healthy volunteers (Zhao et al., 2021). In NSCLC patients, fecal samples showed decreased abundances of Actinobacteria and Proteobacteria, while Firmicutes and Bacteroidetes were increased (Qian et al., 2022). Additionally, NSCLC patients exhibited significantly higher abundances of *Prevotella* spp., *Roseburia* spp., and *Gemmiger* spp. (Qian et al., 2022). In fecal samples from patients newly diagnosed with metastatic NSCLC and lacking driver gene mutations (e.g., epidermal growth factor receptor, anaplastic lymphoma kinase, receptor tyrosine kinase), the top 10 most abundant genera were *Blautia* spp.*, Streptococcus* spp.*, Faecalibacterium* spp.*, Collinsella* spp.*, Bacteroides* spp.*, Dorea* spp.*, Eubacterium hallii* spp.*, Romboutsia* spp.*, Lactobacillus* spp. and *Subdoligranulum* spp. (Li et al., 2024).

In parallel with these microbial disturbances, lung cancer patients display significant perturbations in their gut metabolic profiles. Under normal physiological conditions, dietary carbohydrates metabolized by gut microbiota lead to increased production of SCFAs, which play crucial roles in modulating pulmonary immune responses (Zheng et al., 2020). Of particular importance, Firmicutes and Actinobacteria phyla serve as major contributors to colonic SCFA generation, exerting regulatory effects on inflammatory processes and tumor development in both experimental models and human subjects. The characteristic reduction in Firmicutes/Bacteroidetes ratio observed in lung cancer patients likely contributes to diminished circulating SCFA levels, with consequent impacts on systemic immune function and inflammatory regulation (Zheng et al., 2020). The metabolic imbalance in these patients is further complicated by increased lactate accumulation coupled with SCFA depletion, creating an environment that favors *Candida* proliferation by providing lactate as an alternative energy source, thereby predisposing to fungal infections (Seelbinder et al., 2023). Additionally, enhanced biosynthetic capacity for lipopolysaccharides within the gut microbial community has been mechanistically linked to the development of cancer-associated cachexia in lung cancer patients (Ni et al., 2021). The gut microbiome in NSCLC patients was found to be involved in sporulation and thiamine metabolism (Qian et al., 2022).

## 4 Therapeutic potential of gut microbiota in lung cancer management

### 4.1 Microbial biomarkers for early lung cancer detection

A pivotal case–control study investigating gut microbiome signatures for early lung cancer detection analyzed the fecal microbiota of 42 treatment-naïve early-stage lung cancer patients compared with 65 healthy controls. The research revealed distinct microbial alterations in cancer patients, characterized by significant enrichment of Bacteroidetes and Proteobacteria phyla alongside marked depletion of Firmicutes and Actinobacteria species. Through sophisticated bioinformatics analysis, the study identified a panel of 13 high-specificity microbial biomarkers that demonstrated remarkable diagnostic potential. To translate these findings into clinical practice, the researchers developed a novel Patient Discrimination Index (PDI) algorithm, which achieved outstanding diagnostic performance with an area under the curve (AUC) of 92.4% in the discovery cohort and maintained robust predictive accuracy (AUC = 67.7%) in the independent validation cohort (Zheng et al., 2020). This groundbreaking work establishes the foundation for non-invasive, microbiome-based early detection strategies in lung cancer.

### 4.2 Predicting immunotherapy response through gut microbiota profiling

Accumulating clinical evidence underscores the critical role of gut microbiota composition in determining immunotherapy outcomes for lung cancer patients. Comprehensive analyses demonstrate that baseline gut microbiome characteristics strongly correlate with initial response to immune checkpoint inhibitors (ICIs) during the critical first 6 months of treatment (Gopalakrishnan et al., 2018). A study involving 74 patients with advanced EGFR-mutated NSCLC found that antibiotic use attenuated the efficacy of immunotherapy in these patients, whereas probiotic administration showed no significant impact on treatment outcomes. Furthermore, two dynamic patterns of gut microbiota during immunotherapy were identified: U-shaped and inverted U-shaped trajectories. In the U-shaped pattern, the relative abundance of gut microbiota decreased from baseline to treatment response, followed by an increase from response to disease progression. Conversely, the inverted U-shaped pattern exhibited an initial increase in relative abundance from baseline to treatment response, subsequently declining from response to progression. Significant correlations were observed between gut microbiota/metabolites and immunotherapy response (Luo et al., 2024). Key findings include the striking association between *Akkermansia muciniphila* colonization and superior clinical responses, likely mediated through IL-12-dependent enhancement of anti-tumor immunity (Kaiser, 2017). Furthermore, patients harboring diverse gut microbial communities and enriched populations of *Faecalibacterium* spp. and *Clostridiales* consistently demonstrate improved outcomes with PD-1 inhibitor therapy (Kaiser, 2017). A study analyzing lung cancer patients treated with ICIs revealed distinct baseline gut microbial compositions in advanced patients who derived long-term clinical benefits from ICIs, compared to those with acquired resistance or severe immune-related adverse events (irAEs). Metabolomic profiling demonstrated significantly higher levels of acetate and butyrate in the benefit group versus the resistance group. Patients with elevated acetate, propionate, and butyrate exhibited significantly prolonged progression-free survival (Liu et al., 2024).

A landmark metagenomic study of 245 NSCLC patients employed advanced network analysis to identify two clinically relevant species interaction groups (SIGs): SIG1 (37 species) associated with poor ICI response and SIG2 (45 species) predictive of favorable outcomes. By integrating the SIG1/SIG2 ratio with *Akkermansia muciniphila* abundance, the researchers developed a sophisticated topological scoring system (TOPOSCORE) that accurately predicts individual patient responses to immunotherapy (Derosa et al., 2024). These findings provide a robust framework for personalized treatment selection based on microbial profiling. A multi-omics analysis of fecal microbiota and metabolites was conducted in 303 cancer patients receiving anti-PD-1/PD-L1 immunotherapy, integrated with data from four public metagenomic datasets (568 patients). The study identified five gut microbial enterotypes closely associated with treatment response. Each enterotype demonstrated distinct bacterial compositions and unique metabolic profiles. These enterotypes and their associated metabolites may serve as predictive biomarkers for immunotherapy response (Zhu et al., 2025).

### 4.3 Microbiome-mediated modulation of drug efficacy

The gut microbiome exerts profound influence on anti-tumor drug responses through multifaceted immunomodulatory mechanisms. Beneficial commensals including *Lachnospiraceae, Ruminococcaceae, Faecalibacterium* spp., *Akkermansia* spp.*, and Bifidobacterium* spp. species enhance treatment efficacy through several synergistic pathways: production of immunostimulatory metabolites (SCFAs, L-arginine, inosine, tryptophan); expression of molecular patterns that activate dendritic cells; and induction of TH1-polarized immune responses via IL-12 and type I interferon signaling (Liu et al., 2024). Several studies have demonstrated that probiotic use is associated with improved overall survival and progression-free survival in NSCLC patients (Zhang and Xu, 2023). Antibiotic use may negatively impact prognosis and treatment efficacy in lung cancer patients by altering gut microbiota composition and reducing beneficial bacteria. Multiple retrospective studies have shown that antibiotic exposure correlates with reduced immunotherapy efficacy and poorer prognosis (Zhang et al., 2021). Research indicates that gut microbiota diversity is closely linked to NSCLC patient outcomes, with higher diversity associated with better prognosis. Specific bacterial species, such as *Akkermansia muciniphila* and *Ruminococcaceae*, have been associated with favorable outcomes in NSCLC patients (Del Giudice et al., 2024). Clinical observations reveal that antibiotic administration prior to ICI therapy dramatically reduces median overall survival from 15.3 to 8.3 months (Routy et al., 2018), while specific microbial taxa demonstrate remarkable therapeutic associations. Bifidobacterium species potentiate PD-1 blockade efficacy by activating antigen-presenting cells (Sivan et al., 2015), and butyrate-producing microbes (*Faecalibacterium* spp.*, Roseburia* spp.*, Anaerobutyricum* spp.) significantly improve outcomes through acetyl-CoA-mediated metabolic programming (Liu et al., 2024). These findings are further supported by compelling preclinical evidence showing that fecal microbiota transplantation (FMT) from responding patients can transfer therapeutic responsiveness to germ-free mice (Routy et al., 2018).

### 4.4 Microbiota-targeted therapeutic interventions

Innovative microbiome-modulating strategies are emerging as promising adjuncts to conventional lung cancer therapies. Current approaches for gut microbiome modulation primarily include interventions such as probiotics, prebiotics, FMT, and lifestyle modifications. In recent years, clinical trials have evaluated the efficacy of these microbiome-targeted interventions, particularly in combination with ICIs, providing both biological rationale and clinical feasibility for gut microbiome modulation as a strategy to enhance cancer treatment response (Elkrief et al., 2025). Emerging evidence suggests that specific probiotic supplementation may serve as an “adjuvant” for immunotherapy. For instance, probiotic formulations containing *Bifidobacterium* and *Clostridium butyricum* have demonstrated the ability to enhance ICI efficacy across multiple studies, improving treatment response rates by 10–15%. These probiotics exert their beneficial effects through multiple mechanisms, including immune balance regulation, intestinal barrier enhancement, and production of beneficial metabolites (Jin et al., 2025). Clinical data demonstrate that probiotic supplementation with *Clostridium butyricum* MIYAIRI 588 significantly extends both progression-free and overall survival in advanced NSCLC patients receiving immunotherapy (Tomita et al., 2020). Preclinical models reveal that probiotic co-administration enhances gefitinib’s anti-tumor activity while antibiotic use abrogates its therapeutic effects (Jiang et al., 2024). Cutting-edge research on postbiotic formulations (e.g., MS-20) demonstrates synergistic effects with PD-1 blockade, significantly enhancing CD8 + T cell infiltration and tumor control (Lee et al., 2024).

FMT refers to the process of transplanting functional gut microbiota from healthy donors into recipients’ intestines. The donor microbiota is processed into suspensions or capsules using an intelligent intestinal processing system, with the goal of reconstructing a functionally normal gut microbiome in patients. This restoration enhances both intestinal and systemic immunity, thereby treating intestinal and extraintestinal diseases (Allegretti et al., 2019). FMT from immunotherapy responders to non-responders has shown remarkable capacity to restore treatment sensitivity in both clinical observations and experimental models (Gharaibeh and Jobin, 2019; Zitvogel et al., 2016; Drew, 2024).

Research has demonstrated that dietary interventions can reshape gut microbiota composition, thereby influencing immunotherapy efficacy. A plant-based diet promotes the growth of beneficial bacterial communities and enhances microbial diversity: whole grains and legumes provide prebiotics that stimulate the proliferation of beneficial bacteria such as Bifidobacterium; polyphenols in fresh fruits and vegetables exhibit anti-inflammatory and immunomodulatory effects; and fermented foods serve as excellent natural sources of probiotics. In contrast, animal-based diets (e.g., meat, eggs, and dairy products) may reduce beneficial microbiota and negatively impact the effectiveness of ICIs. Western diets high in fat and sugar may foster microbiota profiles unfavorable for immunotherapy and should be consumed in moderation (Jin et al., 2025; Yannakoulia and Scarmeas, 2024; Allen, 2025; Abdeen et al., 2025). A study by MD Anderson Cancer Center revealed that increased dietary fiber intake improves response to immunotherapy, with the high-fiber diet group achieving an objective response rate (ORR) of 77%, compared to just 29% in the control group (Spencer et al., 2021). These advances underscore the transformative potential of microbiota-targeted approaches in precision oncology.

## 5 Mechanistic insights into gut microbiota-mediated regulation of lung cancer pathogenesis and therapeutic response

The intricate mechanisms through which gut microbiota influence lung cancer pathogenesis and treatment outcomes encompass a multifaceted network of immunological, metabolic, and microbial interactions. Emerging evidence highlights three primary mechanistic pathways: (1) systemic immune modulation through microbial antigen recognition and cytokine signaling, (2) bacterial metabolite-mediated regulation of host metabolism and epigenetic modifications, and (3) microbiome-dependent modulation of inflammatory cascades that shape the tumor microenvironment (Zhu et al., 2023; Fluckiger et al., 2020; Fidelle et al., 2023). These interconnected pathways collectively impact lung cancer initiation, progression, therapeutic response, and long-term prognosis. Research conducted on Lewis lung carcinoma mouse models revealed that compared to the cisplatin-only treatment group, mice receiving combined antibiotics (vancomycin, ampicillin, neomycin) to disrupt gut homeostasis exhibited larger tumors and shorter survival periods. Conversely, the probiotic (*Lactobacillus* spp.)-supplemented group showed smaller tumors and prolonged survival. Mechanistic investigations further demonstrated that antibiotic treatment upregulated vascular endothelial growth factor A (VEGFA) expression while downregulating BAX and CDKN1B expression, consequently attenuating cisplatin’s antitumor efficacy (Zhu et al., 2023; Qiu et al., 2020).

Notable mechanistic findings include the identification of *Enterococcus* spp. phage TMP sequences in fecal samples as predictive biomarkers for favorable immunotherapy outcomes, likely through molecular mimicry between TMP epitopes and the tumor-associated antigen GPD1-L that enhances anti-tumor immune recognition (Fluckiger et al., 2020; Li et al., 2024). Another significant discovery involves *Enterocloster* species-mediated regulation of gut-tumor immune cell trafficking, where microbial modulation of bile acid metabolism leads to downregulation of mucosal addressin cell adhesion molecule-1 (MAdCAM-1) expression in the ileum. This reduction in MAdCAM-1 decreases gut retention of immunosuppressive α4β7 + CD4 + regulatory T cells (Treg17), promoting their migration to tumor sites and consequently influencing PD-1 immunotherapy efficacy (Fidelle et al., 2023). The segmented filamentous bacteria in the gut microbiota can induce the differentiation of Th17 cells, which play a critical role in intestinal immune defense against extracellular pathogens. Conversely, *Clostridium* spp. promote the differentiation of intestinal Treg cells, essential for maintaining immune tolerance and preventing autoimmune responses (Yang et al., 2025). Through their antigens and metabolites, gut microbes interact with immune cells such as dendritic cells, macrophages, and T cells in the intestine, thereby enhancing the generation and function of regulatory T cells (Treg). Treg cells maintain immune tolerance by secreting anti-inflammatory cytokines that suppress inflammatory responses, thereby preventing attacks on self-tissues and harmless substances (Zhou et al., 2025).

Certain beneficial gut microbiota exhibit protective effects against the initiation and progression of lung cancer. Particularly intriguing is the multifaceted role of *Akkermansia muciniphila* in lung cancer biology. Beyond its well-documented gut microbiota-modulating effects, *Akkermansia muciniphila* demonstrates the remarkable ability to translocate systemically and colonize lung tumor tissues, where it restructures the intratumoral microbial ecosystem. More importantly, *Akkermansia muciniphila* exerts profound metabolic reprogramming effects within the tumor microenvironment through modulation of key metabolic enzymes and metabolites. By selectively inhibiting glucose, glutamine, purine, and pyrimidine metabolic pathways in malignant cells, *Akkermansia muciniphila* creates a metabolically unfavorable niche that suppresses tumor growth while potentially enhancing treatment sensitivity. The positive correlation between intestinal *Akkermansia muciniphila* and intratumoral microbes suggests potential translocation of gut bacteria to tumor tissues, thereby influencing the tumor microenvironment. Studies reveal that intestinal *Akkermansia muciniphila* is associated with an enriched consortium of commensal bacteria, including *Eubacterium hallii* and *Bifidobacterium adolescentis* (Zhu et al., 2023; Zhu et al., 2024). Notably, murine model experiments demonstrate that viable *Akkermansia muciniphila* significantly suppresses tumor growth in Lewis lung carcinoma models. Mechanistically, *Akkermansia muciniphila* restores exhausted CD8^+^T cells to cytotoxic subsets, potently activating CD8^+^T cells and synergistically enhancing the efficacy of anti-PD-1 therapy (Derosa et al., 2022). These findings collectively underscore the sophisticated and multi-layered mechanisms through which gut microbiota influence lung cancer biology, offering novel targets for therapeutic intervention and biomarkers for treatment response prediction.

## 6 Discussion

The gut microbiome is increasingly recognized as a key modulator of lung cancer progression and treatment response through the gut-lung axis (Stevens et al., 2025). Research has revealed that alterations in gut microbiota can serve as predictive biomarkers for early-stage lung cancer development and simultaneously modulate the efficacy of immunotherapy (Zheng et al., 2020; Gopalakrishnan et al., 2018). Current studies have demonstrated that targeted modulation of gut microbiota can significantly influence therapeutic outcomes in lung cancer patients (Elkrief et al., 2025).

Emerging research continues to unravel the complex mechanisms by which gut microbes influence tumor immunity and drug efficacy (Zhu et al., 2023; Fluckiger et al., 2020; Fidelle et al., 2023). The primary mechanism involves gut microbiota metabolites influencing lung cancer treatment efficacy. Compared to healthy individuals, lung cancer patients exhibit significant alterations in cellular metabolic pathways, suggesting gut microbiota’s potential in modulating metabolism to suppress tumor growth. Specifically, lung cancer patients show enhanced metabolic activity in: antigen processing, steroid biosynthesis, ubiquitin-mediated protein degradation, transcription factor-related protein activity, bile acid secretion, and mitochondrial fatty acid elongation. Conversely, reduced activity is observed in: bacterial motility proteins, chemotaxis behaviors, flavonoid/flavonol biosynthesis, apoptosis regulation, and G protein-coupled receptor signaling pathways (Qian et al., 2022; Liu et al., 2024; Zhu et al., 2023; Yang et al., 2023).

Additionally, gut microbes and their metabolites regulate host immunity by modulating immune cell migration, activation and function. Studies reveal that gut microbiota and their products locally influence intestinal immunity, causing dysregulation of immune cells and factors, which subsequently affects pulmonary immunity via lymphatic and circulatory systems. Toll-like receptors (TLRs) interacting directly with gut lumen are present not only in intestinal epithelial cells but also in lamina propria immune cells. Microbial products entering the mucosa are phagocytosed and transported by antigen-presenting cells to mesenteric lymph nodes, activating T/B cells. These activated cells then migrate back to lungs via lymphatic/hematogenous circulation, either directly targeting cells or further stimulating other immune components (Jin et al., 2025; Yang et al., 2025; Fofanova et al., 2024; Edwards and Brockmann, 2025).

The gut microbiota has garnered significant attention as a potential adjuvant target for lung cancer therapy. Modulating gut microbial communities to regulate host immune responses and enhance chemotherapy or immunotherapy efficacy has emerged as a novel strategy in precision oncology. Multiple studies demonstrate a strong correlation between gut microbiome composition and the effectiveness of ICIs (Liu et al., 2024; Lin et al., 2025; Lee et al., 2022). For instance, gut microbiota enriched with *Akkermansia muciniphila* and *Bifidobacterium longum* promotes CD8^+^T cell infiltration into the tumor microenvironment, thereby potentiating the anti-tumor effects of PD-1/PD-L1 inhibitors (Zhu et al., 2024; Yan et al., 2023; Nan et al., 2025). Furthermore, microbial metabolites like SCFAs indirectly influence lung cancer progression by regulating Tregs and dendritic cell functions (Ma et al., 2024; Li et al., 2025). Preclinical studies support gut microbiome interventions - including probiotics, prebiotics, or FMT - to improve therapeutic outcomes (Nobels et al., 2025). Mouse models show oral probiotics (e.g., *Lactobacillus* spp.) can mitigate chemotherapy-induced intestinal mucosal damage while enhancing anti-tumor immunity (Sun et al., 2025). FMT trials have also demonstrated that transferring gut microbiota from ICI responders to non-responders can partially restore treatment sensitivity (Gharaibeh and Jobin, 2019; Zitvogel et al., 2016; Drew, 2024). However, clinical translation faces challenges including interindividual variability in microbiome responses, long-term safety concerns, and lack of standardized protocols. Future research should integrate multi-omics approaches (e.g., metagenomics, metabolomics) to identify key microbial species and mechanisms, alongside randomized controlled trials to validate clinical benefits of these interventions.

Key future directions include developing microbiome-based diagnostic tools and targeted modulation strategies to enhance treatment outcomes. Precision approaches like next-generation probiotics and optimized FMT show particular promise for improving immunotherapy responses. However, challenges remain in standardizing methodologies and establishing causal relationships through rigorous clinical studies. As this field advances, integrating microbiome profiling with other omics data will enable more personalized treatment strategies. The coming years will likely see these scientific insights translated into clinical applications, potentially transforming lung cancer management through microbiome-informed approaches.
